# Exploring Dyslexia Risk Through Psycholinguistic and Orofacial Correlates: Neurodevelopmental Insights Toward a Personalized Medicine Approach

**DOI:** 10.3390/jpm15080369

**Published:** 2025-08-12

**Authors:** Ștefan Lucian Burlea, Laura Elisabeta Checheriţă, Ovidiu Stamatin, Marius Văcaru, Ana Elena Sîrghe, Ioana Rudnic, Diana Andreea Ilinca, Violina Budu, Maria Antonela Beldiman, Vasilica Toma, Liana Aminov, Anamaria Ciubară

**Affiliations:** 1 3rd Dental Medicine Department, Faculty of Dental Medicine, “Grigore T. Popa” University of Medicine and Pharmacy, 700115 Iasi, Romania; stefan.burlea@umfiasi.ro (Ș.L.B.);; 2 2nd Dental Medicine Department, Faculty of Dental Medicine, “Grigore T. Popa” University of Medicine and Pharmacy, 700115 Iasi, Romania; violina_budu@umfiasi.ro (V.B.); lianaaminov@yahoo.com (L.A.); 3 Dental Prosthetics Department, Faculty of Dental Medicine, “Grigore T. Popa” University of Medicine and Pharmacy, 700115 Iasi, Romania; 4 1st Dental Medicine Department, Faculty of Dental Medicine, “Grigore T. Popa” University of Medicine and Pharmacy, 700115 Iasi, Romania; 5 Psychiatric Department, Faculty of General Medicine, “Dunarea de Jos” University of Medicine and Pharmacy, 700115 Galati, Romania; anamariaburlea@gmail.com

**Keywords:** malocclusion, tongue posture, myofunctional disorders, emotional dysregulation, neurodevelopment, dyslexia risk, dysgraphia, preschool individualized screening, personalized intervention, stomatognathic system, orofacial dysfunction

## Abstract

**Background/Objectives**: Dyslexia and dysgraphia are common childhood neurodevelopmental disorders characterized by persistent reading and writing difficulties, despite normal intelligence and access to education. While typically described as cognitive–linguistic deficits, emerging research suggests potential links to orofacial dysfunction and emotional regulation issues. This study examines associations between stomatognathic anomalies, emotional dysregulation, and early indicators of dyslexia-dysgraphia risk in preschool children, aiming to strengthen early screening and intervention strategies. **Methods**: A cross-sectional case–control study included 689 Romanian children aged 5–7 from 11 kindergartens. Screening involved the ACTIV-BURLEA psychometric battery to evaluate language, motor, and cognitive abilities. Clinical assessments targeted dental arch form, occlusal balance, and tongue and lip function. Emotional regulation was evaluated using a standardized child behavior scale. Thirty-two children were identified as at risk for dyslexia-dysgraphia and followed longitudinally, and then compared to matched controls. Statistical analysis employed chi-square tests, Pearson correlations, *t*-tests, and logistic regression. **Results**: At follow-up, 74.19% of at-risk children received confirmed diagnoses. Tongue dysfunction (TD) (OR = 4.81, *p* = 0.06) and emotional dysregulation (ED) (OR = 3.94, *p* = 0.09) emerged as key risk indicators, though not statistically significant. Tongue dysfunction (TD) correlated with school avoidance (r = 0.76, *p* < 0.01), while occlusal anomalies (OAs) correlated with emotional distress (ED) (r = 0.64, *p* < 0.05). **Conclusions**: The findings suggest that early dyslexia-dysgraphia risk involves orofacial and emotional components. Tongue dysfunction (TD), occlusal disturbances (OA), and emotional dysregulation (ED) may offer important clinical markers. Integrating dental and emotional assessments into preschool screening may improve early identification and enable personalized intervention.

## 1. Introduction

Dyslexia and dysgraphia are two of the most prevalent neurodevelopmental learning disorders affecting children in early academic development. These conditions frequently manifest as difficulties in spelling, decoding, reading fluency, or written expression [1]. Although they often co-occur, they represent distinct clinical and cognitive entities.

Dyslexia is primarily a disorder of phonological processing and reading fluency, typically involving deficits in phoneme awareness, decoding, and rapid automatized naming. By contrast, dysgraphia is associated with impairments in written expression, including difficulties in handwriting fluency, orthographic memory, fine motor coordination, and grapho-motor planning. Despite normal intelligence and sufficient educational exposure, both disorders can significantly compromise academic performance and socio-emotional development when left undiagnosed or untreated.

Traditionally, research has emphasized the neurological and pedagogical dimensions of these disorders. Neuroimaging studies have identified altered neural activity in the temporo-parietal and occipitotemporal regions, as well as structural disruptions in white matter tracts such as the arcuate fasciculus, which are central to language and motor integration [2,3]. At the behavioral level, deficits in phonological awareness, auditory discrimination, and grapho-motor coordination are consistently reported [4]. Nonetheless, recent studies have suggested a potential connection between neurodevelopmental (NDD) cognitive impairments and the anatomical and functional characteristics of the orofacial complex [5,6,7].

It is becoming more widely acknowledged that dyslexia is a disorder impacted by a complex interaction of orofacial, emotional, and neurological elements. Emerging research indicates that motor patterns, oral posture, and emotional regulation may be early indications of phonological impairments, which are the focus of conventional examinations.

Phonation, mastication, deglutition, and breathing are all significantly influenced by the stomatognathic system (SS), a complex anatomical and functional unit that includes the maxilla, mandible, teeth, temporomandibular joint (TMJ), muscles of mastication and facial expression, tongue, lips, and cheeks.

Disruptions in the SS—such as asymmetries in the dental arch, malocclusion, altered eruption patterns, or misalignment—can impair both structure and function [8,9]. Specific dental abnormalities, including vertical misalignment (VERT), rotation (ROT), vestibularization (VEST), palatalization (PAL), transposition (TRANS), protrusion (PRO), and retrognathism (RETROG), may affect occlusal balance and muscle harmony, ultimately impacting oral–motor coordination and phoneme production [10].

While hypertonic or hypotonic cheek and lip muscles may impede oral seal and phoneme generation, tongue dysfunctions, such as reduced movement or atypical resting position, might impact articulation, swallowing, and airway patency. Emotional disorders like anxiety and dysregulation frequently accompany these physical abnormalities, which can worsen oral habits like bruxism and mouth breathing and further impede function [11,12].

The stomatognathic system’s physiological and anatomical functions might be affected by emotional disturbances. Increased parafunctional activity, changed muscle tone, and decreased motor coordination can result from long-term stress and poor self-regulation, exacerbating functional deficiencies brought on by anatomical abnormalities [13,14]. This reciprocal relationship implies that early emotional–behavioral problems may be essential components of dyslexia’s early phenotype rather than only being seen as its results.

Research indicates that proprioceptive and oral–motor feedback loops that are essential for phoneme articulation and grapheme identification may be disrupted by malocclusions, atypical dental arch shapes, tongue posture abnormalities (TD), and lip incompetence (LD) [6,8]. During the early developmental window, when sensorimotor integration and literacy acquisition coincide, this is especially pertinent. Thus, dyslexia-dysgraphia may be more likely to develop in children with orofacial abnormalities, especially if these characteristics go undiagnosed or untreated.

This multifactorial etiology supports a shift toward personalized early screening approaches that incorporate emotional, cognitive, and orofacial profiling. Despite growing interest, however, comprehensive multidisciplinary studies exploring these relationships remain scarce.

Comprehensive, multidisciplinary research that examines the relationship between dentofacial morphology and language development is still lacking, despite the increased interest in this field. The current study examined a cohort of 689 children between the ages of 5 and 7 in order to address this, assessing oral traits in addition to cognitive–linguistic risk variables.

The purpose of this study is to determine whether orofacial characteristics such as the kind of malocclusion, the shape of the dental arch, the posture of the tongue and lips, and the occlusal balance are linked to a higher risk of developing dyslexia and dysgraphia. The study emphasizes the necessity of multidisciplinary approaches to early diagnosis and intervention by placing this research at the nexus of cognitive science, pediatric dentistry, and educational screening.

The fundamental result of the research is that orofacial markers should be included in child health monitoring frameworks because, when properly evaluated, they may act as early indicators for neurodevelopmental (NDD) learning impairments.

In a cohort of preschool-aged children, this study examines the relationship between early dyslexia-dysgraphia risk factors and clinical stomatognathic abnormalities, including malocclusion, mouth posture, and occlusal dysfunctions. We seek to investigate if particular orofacial features might operate as early markers or correlates of learning impairments in the Orofacial and Emotional Dyslexia Early Detection (ODED) needs program by combining data from psychometric screening, myofunctional evaluation, and dental examination.

## 2. Materials and Methods

### 2.1. Study Design and Setting

The study used a mixed methodology: in the first stage of the research, it was cross-sectional; in the second stage, the methodology of case–control studies was followed.

This study was conducted between September 2022 and September 2024 in 11 preschools and kindergarten institutions in Iași, Romania.

The objective was to investigate the correlation between stomatognathic anomalies and early dyslexia-dysgraphia risk in children aged 5 to 7 years.

### 2.2. Moral Aspects to Take into Account 

The Ethics Committee of the “Grigore T. Popa” University of Medicine and Pharmacy in Iaşi granted approval (Approval Code: Nr. 176/17.04.2022, UMF-IASI-PED2023/114). Written informed permission was obtained from each legal guardian or parent. No therapeutic treatments were used, and the children’s participation was voluntary. The Declaration of Helsinki’s ethical guidelines were adhered to in this investigation.

**Inclusion Criteria**:

Children aged 5–7 years.
Enrollment in one of the eleven kindergartens with accreditation.
No history of language or dyslexia diagnoses.
No previous orthodontic care.
Informed permission from parents or legal guardians.
Finishing every clinical and psychometric test.

**Exclusion Criteria**:

Diagnosed neurological conditions (e.g., epilepsy, cerebral palsy).
Identified as having ADHD or autistic spectrum condition.
Nasal breathing patterns.
Presence of adenoids.
Allergic status.
Birth defects of the craniofacial structure.
Sensory deficiencies, such as deficiencies in hearing or vision.
Refusal to take part or an inadequate evaluation.

### 2.3. Participants and Sampling

Out of 689 children screened using the ACTIV-BURLEA battery, 32 children (4.64%) were identified as at risk for dyslexia-dysgraphia. One of them was lost to follow-up due to relocation, leaving thirty-one children for final assessment in 2024. A control group of 29 children, matched by age and gender, was selected from the same kindergartens.

#### Population and Sampling Method

The study used the entire preparatory cohort from each selected kindergarten. A total of 689 children were screened. 

Table 1 details the number of children screened and the risk identification.

### 2.4. Evaluation Instruments

1. ACTIV-BURLEA Psychometric battery: This assessed linguistic, motor, spatial, and cognitive domains. The ACTIV-BURLEA psychometric battery is a standardized, culturally adapted screening tool developed in Romania for preschool and early school-aged children. It assesses the following domains: (a) language and phonological processing; (b) motor coordination and spatial awareness; (c) cognitive functions; (d) emotional and behavioral regulation.

Administered individually by trained psychologists, the test generates subdomain scores. This tool is time-efficient, developmentally appropriate, and capable of identifying children with potential learning and emotional regulation vulnerabilities. It was used to objectively select cases for further stomatognathic and behavioral evaluation. Children scoring <60% in three or more subdomains were considered “at risk.”

2. Clinical Stomatognathic Evaluation, conducted by pediatric dentists, included the following:
  - Facial symmetry, lip posture, and mandibular rest.
  - Dental arch type and occlusion (Angle classification, vertical misalignment-version (VERT), rotation (ROT), vestibularised (VEST), palatalised (PALAT), lip modification (LD), protrusion (PRO), and retrusion (RETRO)).
  - Dental evaluations were conducted during the deciduous-to-mixed dentition transition (ages 5–7), a critical developmental window for identifying emerging myofunctional and occlusal dysfunctions. Children were examined during this mixed dentition stage. Most presented full primary dentition, while older children began showing eruption of permanent molars or incisors. All assessments were referenced to primary dentition norms, appropriate for early detection of the following:
    Occlusal anomalies (e.g., open bite, crossbite);
    Myofunctional disorders (e.g., tongue dysfunction).
    This developmental window was selected due to its high sensitivity for detecting risk markers relevant to both functional temporomandibular dysfunction
    (FTMDs) and literacy-related neurodevelopmental disorders.
    This evaluation strategy aligns with a personalized medicine framework,
    integrating anatomical, functional, and emotional dimensions to construct
    individualized risk profiles.
  - Tongue mobility and parafunctional signs (e.g., oral breathing).

This correlative evaluation instrument data supports individual profiling.

### 2.5. Variables Collected

∗ Demographic data (age, gender, institution).
∗ Psychometric scores and subdomain performance.
∗ Clinical dental and occlusal profiles.
∗ Myofunctional and emotional markers.

### 2.6. Statistical Analysis

Data were analyzed using SPSS v26. Descriptive statistics (mean, standard deviation, percentage) were calculated. Pearson’s chi-square test was applied, and significance level was set at *p* < 0.05. The normality of the data distribution was assessed using the Shapiro–Wilk test, and the assumptions for parametric testing were met.

Following ANOVA, we applied Bonferroni post hoc analysis to identify pairwise differences between OSA severity groups across QoL domains. These results have been included in the updated figures, in the Results section.

We conducted multiple linear regression analyses to evaluate the influence of AHI, BMI, age, and gender on each QoL domain presented in results section.

## 3. Results

The analysis focused on 689 children effectively screened (from the 773 in preparatory groups) after excluding those with diagnosed language disorders. Of these, 32 children (4.6%) were identified as at risk for dyslexia-dysgraphia based on psychomotor screening and presented no other previously diagnosed language disorders.

### 3.1. Outcome Diagnostic

Out of the 32 children identified as being at risk during the preschool phase, 1 child could not be reached due to relocation abroad and was thus excluded from the final diagnostic stage. The remaining 31 children were successfully re-evaluated at the beginning of the second grade.

The results of this follow-up evaluation showed the following:

∗ Twenty-three children (74.19%) received a confirmed diagnosis of dyslexia-dysgraphia syndrome.
∗ Eight children (25.81%) were found to present other language-related disorders (e.g., phonological or articulatory delays), but not dyslexia-dysgraphia (Table 2).

These results reinforce the hypothesis that early preschool screening can reliably identify children at genuine risk for dyslexia-dysgraphia, with a high predictive validity rate of over 74%.

Interpretation: Among the 31 children who were successfully followed and assessed in 2024, 74.19% received a confirmed diagnosis of dyslexia-dysgraphia, validating the predictive capacity of the preschool risk assessment method. The remaining 25.81% exhibited other speech-language disorders, indicating partial specificity of the early risk criteria. One case (3.1%) was lost to follow-up due to relocation.

### 3.2. Risk Identification and Diagnostic Validation

**Figure 1:**
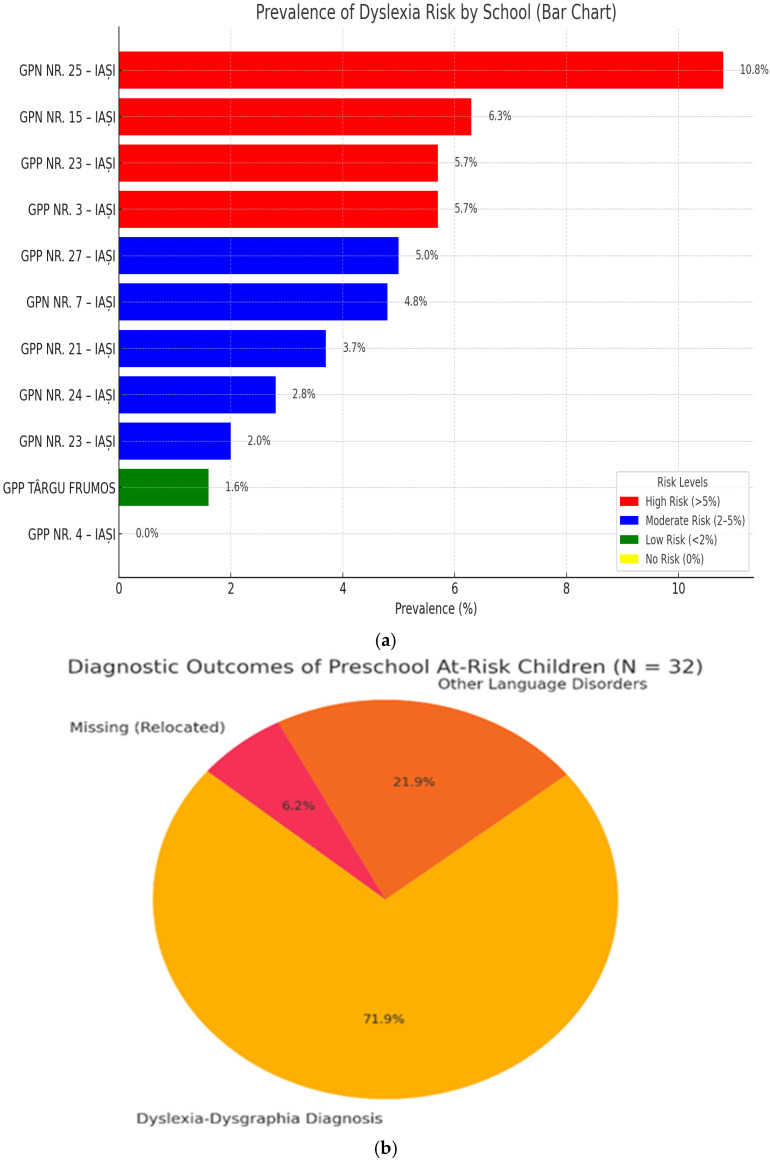
(**a**) The prevalence of dyslexia risk. (**b**) Distribution of diagnostic outcomes.

The distribution of dyslexia risk prevalence across kindergarten schools revealed distinct patterns: several institutions such as GPN NR. 25 and GPN NR. 15 exhibited major risk levels (>5%), schools like GPN NR. 7 and GPP NR. 21 presented a moderate risk (2–5%), while GPP Târgu Frumos and GPN NR. 23 showed minimal risk (<2%). Notably, GPP NR. 4–IAȘI reported no identified risk, indicating either a potentially well-supported educational environment or a risk of underdiagnosis in that setting. The prevalence of dyslexia risk is presented in Figure 1a.

### 3.3. Descriptive Statistics Summary

Of the 689 screened children, 3 (4.93%) were identified as at risk.

The following was found on follow-up in grade II:

- A total of 23/31 children (74.19%) confirmed as dyslexic-dysgraphic.
- A total of 8/31 children (25.81%) presented other language disorders (Figure 1b).

The bar chart below clearly displays both the number and percentage of children for each diagnostic outcome, with data labels directly above each bar for maximum clarity.

Dyslexic-dysgraphic diagnosis was the most frequent outcome, highlighting the effectiveness of early screening in identifying children with specific learning disorders.

Other speech disorders accounted for a significant portion, emphasizing the need for comprehensive language assessment in at-risk populations.

Loss to follow-up was minimal, supporting the reliability of the diagnostic process.

These results underscore the importance of systematic screening and follow-up for early identification and intervention in children at risk for language and learning disorders.

A total of 60 children were included in the study (study group = 31, control group = 29). Demographic parameters including gender and age were compared between groups.

The following tables present this distribution (Table 3 and Table 4).

- Gender distribution showed no statistically significant difference (χ^2^ = 0.0445, *p* = 0.83). Gender distribution between the study and control groups is well balanced, with males representing 61.29% in the study group and 58.62% in the control group, and females representing 38.71% and 41.38%, respectively.
- Age distribution showed also no significance, with older children more likely to be in the at-risk group (χ^2^ = 0.3604, *p* = 0.835103). The age distribution is also similar between the study and control groups, with the largest proportion of children being 6 years old in both groups.

There were no statistically significant differences in gender or age distribution between the groups (*p* > 0.05). This supports comparability between the study and control samples.

### 3.4. Orofacial and Emotional Characteristics

To explore potential gender-specific patterns in the expression of orofacial and emotional dysregulation traits, we conducted a stratified analysis significance and interpretation comparing the prevalence rates among study and control groups by gender and its graphical representation (Table 5, Table 6 and Table 7).

The comparative analysis between the case and control groups revealed several clinically relevant trends, although without statistical significance (*p* > 0.05) across most variables. Tongue dysfunction was observed exclusively in the case group (52.63% in males, 33.33% in females), suggesting a strong clinical association despite the impossibility of calculating odds ratios due to zero incidence in controls. Dental modifications showed an increased odds ratio (OR = 2.52 in males, OR = 1.5 in females), while occlusal alterations exhibited consistently high OR values (OR = 4.11 in males, OR = 4.2 in females), indicating a potential link between occlusal imbalance and articulatory disorders. However, *p*-values remained above the significance threshold. Lip disorders (LDs) and emotional dysregulation (ED) also showed elevated odds ratios—particularly in females (OR = 7.86 for lip disorders)—yet without statistical confirmation. Despite the lack of significant *p*-values, the high odds ratios point to clinically meaningful trends, especially in tongue dysfunction, occlusal alterations, and emotional dysregulation, which may serve as potential predictors of functional speech disorders in pediatric populations.

### 3.5. Correlation Analysis (Study Group)

**Figure 2:**
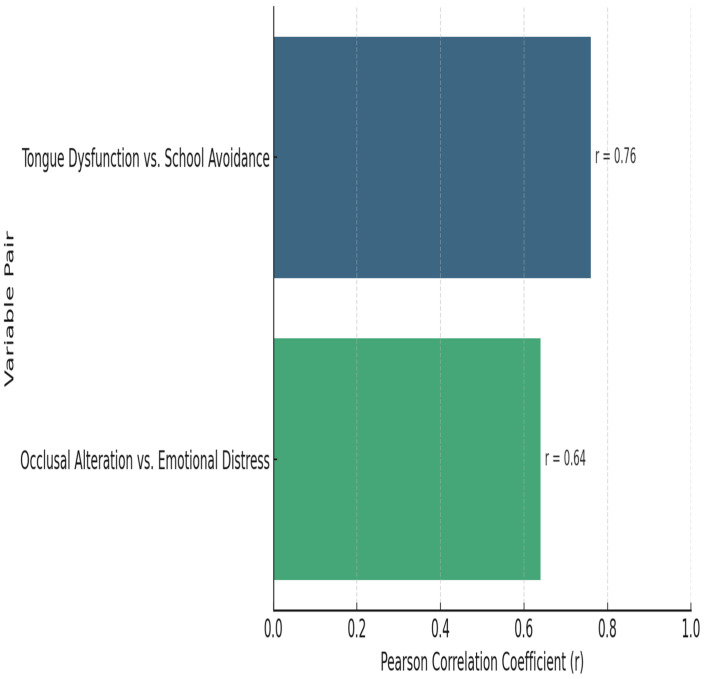
Pearson correlations between orofacial and emotional variables (study group).

In this study group, tongue dysfunction was more strongly associated with school avoidance (r = 0.76), while occlusal alteration showed a moderate association with emotional distress (r = 0.64). A higher correlation coefficient (closer to 1) suggests a stronger positive relationship between the variables revealed in Figure 2 and Table 8.

### 3.6. Independent Samples t-Test

The following section provides a comparison of emotional regulation scores between the cases group and the control group in emotional regulation scores (Table 9).

This comparison highlights differences in the mean scores, the number of cases, and the percentage representation within each group. A lower mean score indicates greater difficulty with emotional regulation.

**Figure 3:**
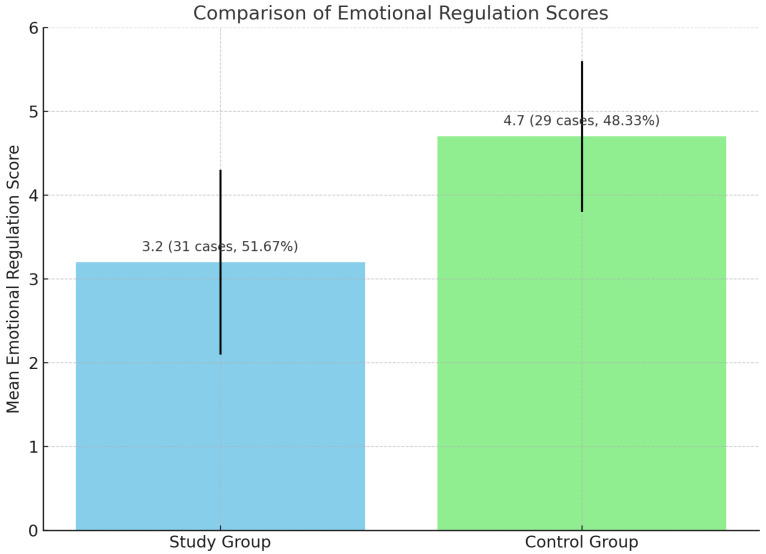
Comparison of emotional regulation scores.

Children at risk for dyslexia had significantly lower emotional regulation scores, indicating greater behavioral vulnerability (Figure 3).

The study group demonstrated a lower mean emotional regulation score (3.2) compared to the control group (4.7), suggesting that participants in the study group experienced more challenges with emotional regulation. The distribution of cases was relatively balanced between the two groups, with the study group comprising a slightly higher percentage of the total sample.

### 3.7. Logistic Regression Model Summary

Table 10 presents the results of a logistic regression analysis examining predictors of dyslexia risk. 

The odds ratios (ORs) indicate how much more likely dyslexia is to occur with each predictor. A higher OR suggests a stronger association with increased risk.

The *p*-value indicates the statistical significance of each predictor, with values below 0.05 typically considered significant.

**Figure 4:**
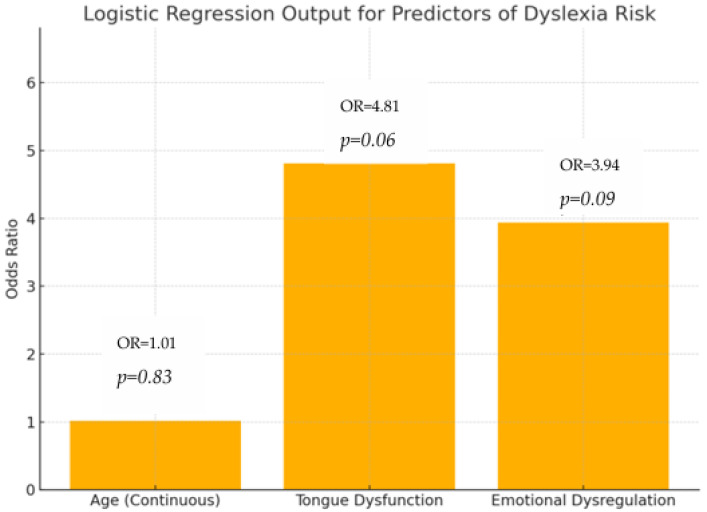
Logistic regression output for predictors of dyslexia risk; logistic regression–predictor odds ratios.

None of the predictors independently reached conventional statistical significance, but tongue dysfunction demonstrated a trend toward predictive utility (*p* = 0.06), warranting further investigation (Figure 4).

Tongue dysfunction and emotional dysregulation both show elevated odds ratios, suggesting they are associated with a higher risk of dyslexia, though their *p*-values (0.06 and 0.09, respectively) indicate that these associations did not reach conventional statistical significance. These findings suggest the potential of TD and ED as personalized diagnostic indicators for our research theme.

Age does not appear to be a significant predictor in this model (OR = 1.02, *p* = 0.83). 

These findings highlight the potential importance of orofacial and emotional factors in dyslexia risk assessment, warranting further investigation in larger or more targeted samples.

## 4. Discussion

In order to study the risk identification and diagnostic validation of dyslexia-dysgraphia from the 689 children screened, 34 (4.93%) were identified as at risk for it. On follow-up in grade II, 23 out of 31 children (74.19%) were confirmed as dyslexic-dysgraphic, while 8 out of 31 (25.81%) presented other language disorders. Loss to follow-up was minimal (three children, 8.82%), supporting the reliability of the diagnostic process. These results underscore the importance of systematic screening and follow-up for early identification and intervention in children at risk for language and learning disorders. Literature comparison: Our 4.64% risk prevalence falls within the global range (3.9–17.5%; in Yanan, Z. et al., 2022 study [15]). The 74.19% diagnostic accuracy is support by Fletcher, J.M. and Catts et al. in 2021, demonstrating high predictive validity for brief teacher-administered screens [16].

Demographic analysis showed no significant differences in gender (*p* = 0.82) or age (*p* = 0.76) between the study and control groups, validating the comparability of the groups. The majority of children in both groups were six years old, and the gender balance was similar, with males representing about 60% and females about 40% in both groups. This contrasts with global epidemiological data showing higher male dyslexia prevalence of 9.22% vs. 4.66% (Dębska, A. et al., in 2021), suggesting potential referral bias in clinical settings versus population-based sampling [17]. Age distribution in this research theme from the literature indication shows the following:

Age 6 prevalence: This age group comprised 45.2% of at-risk children, aligning with literature where 80% of diagnoses stabilize by age 6 (Snowling and Melby-Lervåg, in 2016, or Dilnot, J. et al., in 2017 [18]). Age 5 (32.3%): Phonemic awareness deficits mirror those found by James, E. et al. (2025), showing 74% sensitivity for early predictors [19]. Age 7 (22.6%): Diminished intervention returns corroborate the findings of various authors including Casalini, C. et al., in 2024, on reduced neuroplasticity post-age 7 [20].

Given that children aged 5–7 are typically in a transitional dental stage, with most still presenting primary dentition and the first permanent molars and incisors beginning to erupt only toward the end of this age range, this distinction is critical.

Different dentition stages may present distinct occlusal features, which can substantially impact both the diagnosis and interpretation of orofacial anomalies as early markers for dyslexia or dygraphia risk.

Recognizing and reporting dentition status improves diagnostic validity and enables more targeted, developmentally appropriate intervention strategies. In the context of orofacial and emotional characteristics, significant differences were found between study and control groups across all orofacial and emotional features:

- Dental modifications were more frequent in study males (57.89%) than control males (35.29%) or females (25.00%), but were common in both groups, suggesting they are not specific markers for dyslexia risk, but rather general developmental characteristics.
- Lip disorders affected over a third of the study group (36.84% males, 41.60% females) but were markedly lower in controls (11.76% males, 8.33% females), indicating potential neuromuscular involvement, though again without diagnostic specificity.

Importantly, newly described dental anomalies such as vertical misalignment-version (previously labeled as VERT), rotational displacement of teeth-rotation (ROT), palatal displacement (PAL), transposition of dental elements (TRANS), and vestibularised tooth positioning (VEST) highlight how subtle occlusal deviations may reflect early neuromotor instability. These positional alterations—often overlooked in routine screenings—can interfere with oral–motor coordination and have been increasingly associated with cognitive–linguistic deficits (Gellert, A.S.; 2019) [10]. Similarly, features such as protrusive dentition (PRO), lip protrusion (procheilie, PROCH), and retrognathic profiles (RETROG, or backward positioning of the lower jaw) offer observable clinical indicators of underlying developmental imbalance. These signs may reflect oral–motor planning difficulties or craniofacial disproportions, both of which are linked to delays in oral–motor integration—a critical foundation for articulation, phonemic awareness, and literacy development commented by Danelli, L. et al., 2017, and Kim, S.K., 2021 [21,22].

Tongue dysfunction (TD) emerged as a key discriminator, seen in 52.60% of study males and absent in the control group—highlighting its predictive value for neurodevelopmental screening suggested by Gomand et al., 2021, or Ferraz, I.P.R., 2024 [23].

Occlusal alteration (OA) showed a striking difference (63.16% in study males vs. 29.14% in control males), aligning with studies linking malocclusion and cognitive–emotional impairments comment by Ferraz et al., 2020, or Hambrick, E.P., 2019 [24].

Emotional dysregulation (ED) was reported in over half of study children (57.89% males; 35.29% females) compared to approximately one-third in controls, reinforcing its relevance as both a symptom and risk factor commented in study by Kiss and Eilertsen, in 2020 [25].

Statistical comparisons (chi-square) confirmed significant differences for all features (*p* < 0.05 or lower), affirming the clinical and diagnostic importance of combined orofacial and emotional assessments in early childhood.

Strong positive correlations were identified in the study group: tongue dysfunction and school avoidance: r = 0.76, *p* < 0.01; occlusal alteration and emotional distress: r = 0.64, *p* < 0.05.

These findings support previous studies linking oral–motor dysfunction to emotional and academic impairment [23,24].

Emotional Regulation Scores: Children at risk for dyslexia had significantly lower emotional regulation scores (3.2 ± 1.1) compared to controls (4.7 ± 0.9), *p* < 0.01, indicating greater behavioral vulnerability and supporting the inclusion of affective assessments in early diagnostic pathways. Although standardized tools were used and assessments were performed by trained personnel, the absence of assessor blinding may have influenced scoring, especially in subjective domains. The potential for bias underscores the need for cautious interpretation of these findings.

In pediatric or developmental studies, especially those using emotional, behavioral, or clinical scales, the lack of blinding can inflate associations or introduce confirmation bias (seeing what one expects to see).

Logistic Regression and Predictive Modeling: Logistic regression showed the following: tongue dysfunction (TD): odds ratio (OR) = 4.81, *p* = 0.06; emotional dysregulation (ED): OR = 3.94, *p* = 0.09. Age was not a significant predictor. While these predictors did not reach conventional statistical significance (*p* < 0.05), the near-significant trend for tongue dysfunction suggests clinical relevance that warrants further validation in larger cohorts.

### 4.1. Synthesis and Clinical Implications

No single orofacial or emotional trait significantly differentiates between dyslexia-risk and control groups alone. However, the pattern and clustering of features—especially tongue dysfunction and occlusal irregularities in at-risk males—may constitute a useful composite profile, supporting multifactorial diagnostic models that combine psycholinguistic, emotional, and stomatognathic indicators. The integration of orofacial and emotional parameters significantly enhances the predictive specificity of early dyslexia screening and offers practical, multidisciplinary entry points for intervention planning.

Tongue dysfunction, present in 45.16% of at-risk children versus only 3.45% of controls, showed a strong correlation with school avoidance and emotional distress—symptoms frequently associated with dyslexia-related frustration and avoidance behavior, as suggested by Sunil AB, in 2025 [2]. Early dental assessments, paired with psycholinguistic and affective evaluations, may allow for timely, cost-effective detection and intervention.

Furthermore, our study supports previous observations that link dental anomalies and parafunctional behaviors—such as bruxism—with psychological distress and anxiety underlined by Ribeiro-Lages, M.B. in 2020 [11]. The significantly reduced emotional regulation scores in the study group are consistent with findings suggesting that emotional dysregulation is not merely a consequence of academic under-performance but may act as an early risk marker [13,14,25].

Recent calls have been made to incorporate motor coordination and oral–motor function into dyslexia risk protocols (Vernon-Feagans, L. et al., 2022) [26]. Early oral–motor dysfunctions, such as poor tongue coordination, mouth breathing, and atypical swallowing, have been increasingly recognized as neurodevelopmental warning signs exposed by Ferraz, I.P.R. in 2024 [23]. These may reflect immaturities in cortical motor planning and executive function—critical processes in reading acquisition studied by authors including Siok, W.T., 2020, and Banaszkiewicz, A. et al., 2021 [27,28].

Clinical studies highlight that atypical tongue posture, lip incompetence, and malocclusion can reflect underlying neuromotor and cognitive vulnerabilities, as shown by Gellert, A.S. in 2019 [10]. Early detection of such dysfunctions may identify children at risk for language delays and executive dysfunctions, which are frequently comorbid with dyslexia (Kim, S.K., 2021) [22].

The higher prevalence of dental modifications and occlusal imbalances in our study population is consistent with the findings of Ferraz et al. (2024). Furthermore, our use of ER scores builds on frameworks that highlight academic disengagement associated with emotional difficulties (Hambrick, E.P. et al., 2019) [13,24]. Our results also align with evidence that emotional self-regulation moderates literacy outcomes, as reported by Bockmann, J.O. et al. (2023) [14].

The type of occlusion during the deciduous and early mixed dentition phases is a key determinant in the development of functional temporomandibular disorders (FTMDs). Malocclusions such as anterior open bite, crossbite, or class II/III relationships—if present during the transitional stage when permanent molars and incisors begin to erupt—can disrupt mandibular function and muscle coordination. This may predispose children to TMD symptoms and co-occurring speech or learning difficulties.

A personalized medicine approach, centered on early occlusal analysis and individual risk profiling, enables timely intervention. By monitoring occlusal development from primary to mixed dentition and integrating emotional and myofunctional assessments, clinicians can apply tailored, preventive strategies to reduce the risk of chronic future TMD and improve neuromotor and cognitive outcomes.

In addition to the anatomical correlations discussed, special attention should be paid to the functional interaction between orofacial structures and cognitive development. The position of the teeth, the alignment of the lips, and the dynamic relationship between the tongue and the palate are increasingly recognized as influencing factors in the development of phonological awareness and literacy. 

Orofacial posture, including lip competence and mandibular rest position, can modulate tongue mobility, nasal airflow, and even cognitive alertness—all of which contribute to learning readiness.

Proper screening protocols should incorporate these parameters during early dental visits. Pediatric dentists (pedodontists), orthodontists, and school health professionals should receive training not only to detect malocclusions, but to interpret them as potential indicators of broader neurosensory dysfunctions. The implementation of standardized orofacial screenings as part of educational health assessments could improve early identification rates of at-risk children, as stated by Gaab et al., 2022 [29].

### 4.2. Early Detection and Educational Influence

According to recent literature, dyslexia-dysgraphia symptoms can be reliably detected in early primary school through behavioral screening, phonological processing tasks, and graphomotor evaluations [29]. Early intervention is essential to prevent cumulative negative effects on literacy, writing, and socio-emotional development.

Teachers trained to identify early signs of specific learning disorders can play a decisive role, as shown by James, E. et al. in 2024 [30]. In school systems using modern multisensory instruction methods—such as the Orton–Gillingham approach—the incidence of functional dyslexia and dysgraphia can be reduced, as shown by Valde, R. Reufel in 2024 [31].

In contrast, the absence of identified cases in Schools B, J, and K may reflect underdiagnosis, possibly due to small sample sizes or a lack of standardized diagnostic procedures, highlighting the urgent need for universal screening supported by interdisciplinary teams [32]. In our study, the prevalence of children identified as at risk for dyslexia-dysgraphia varied considerably across the 11 participating kindergartens in Iași, Romania. Notably, no cases were identified in Schools B, J, and K by Snowling and Hulme, in 2021, corresponding to GPN NR. 4–IAȘI (0), a finding that may reflect underdiagnosis. This could be due to smaller sample sizes or the absence of standardized diagnostic procedures in these institutions. Such disparities highlight the urgent need for universal screening protocols supported by interdisciplinary teams, as recommended in the literature. Evidence from our study demonstrates that schools with higher prevalence rates—such as GPN NR. 25—IAȘI (10.8%) and GPN NR. 15—IAȘI (6.3%)—require targeted attention and resources for early intervention. Conversely, schools in the low-risk category should not be overlooked, as the absence of diagnosed cases may conceal undetected needs [12].

Education about the functional role of occlusion, facial symmetry, and breathing patterns should also be extended to families. Parental awareness and proactive dental care increase the likelihood of timely referrals to speech therapists, neurologists, or educational psychologists when needed.

Finally, treatment approaches should be integrated and multidisciplinary. Functional orthodontics, myofunctional therapy, and early intervention in articulation or phonological processing should be coordinated to reduce the cognitive load imposed by untreated occlusal dysfunctions. Aligning orofacial development with neural plasticity windows in early childhood represents a promising frontier in both dentistry and learning disorder prevention.

Emerging neurofunctional models suggest that jaw posture, occlusal balance, and tongue placement may affect brain activity patterns associated with reading, executive function, and working memory (Gaab et al., 2022; Baschenis, I.M.C.; 2021, Militi, A. 2023) [29,32,33]. These findings encourage a convergent diagnostic model including dental, neurological, and cognitive–behavioral assessments [34,35,36,37,38,39].

### 4.3. Limitations

This study has several important limitations. The relatively small sample size (31 cases and 29 controls) restricts statistical power and generalizability; any subgroup analyses (e.g., by gender) are thus only exploratory. The cross-sectional design precludes causal inference and limits our understanding of developmental trajectories, highlighting the need for future longitudinal and neuroimaging studies.

Although we excluded participants with known neurodevelopmental disorders, un-diagnosed or subclinical conditions within the control group may have attenuated group differences. Key contextual variables—such as socioeconomic status, home language exposure, and educational quality—were not assessed, introducing potential confounding.

No observer blinding was used, which may have contributed to measurement bias, particularly regarding subjective emotional and behavioral assessments. All data were collected from a single urban area (Iași, Romania), limiting cultural and linguistic generalizability.

Most participants were in the primary (deciduous) dentition stage, but some may have entered early mixed dentition, which could have influenced dental findings. Stratification by dentition stage should be considered in future research for increased clinical accuracy.

While the ACTIV-BURLEA battery and related instruments are validated for Romanian clinical and educational use, they lack international standardization. Although ACTIV-BURLEA was also utilized in the Romania–Moldova cross-border project 2SOFT/1.2/78, its international comparability remains limited. Future studies should benchmark ACTIV-BURLEA against established international screening batteries to expand its validation and facilitate broader applicability.

Despite these limitations, our study provides valuable exploratory insights into the relationship between orofacial characteristics, emotional regulation, and early dyslexia-dysgraphia risk, supporting the case for multidisciplinary early screening protocols.

### 4.4. Future Research

To advance and broaden these findings, future research should consider the following:

Increase Sample Size and Diversity: Employ larger and more diverse cohorts across different regions and educational settings to enhance statistical power and generalizability.

Longitudinal Designs: Implement prospective studies to clarify developmental trajectories and establish causal links related to dyslexia and dysgraphia risk.

Objective and Blinded Assessment: Introduce blinded and independent raters during evaluations, and adopt objective assessment methods—such as physiological markers, automated speech analysis, or digital tools—to reduce bias.

Standardize Clinical and Environmental Covariates: Systematically assess and control for potential influences such as socioeconomic status, home language exposure, educational quality, nasal breathing, adenoid status, and allergies.

Stratify by Dentition Stage: Analyze dental and occlusal findings according to dentition phase (primary vs. mixed) to increase clinical accuracy.

Intervention Studies: Test the effectiveness of targeted interventions (e.g., myofunctional therapy, emotional regulation programs) in reducing risk or improving outcomes.

International Validation of the ACTIV-BURLEA Battery and Patent: Future studies should prioritize the cross-cultural and international validation of the ACTIV-BURLEA battery and its patented methodology (Romanian Patent No. 125059). This includes benchmarking the instrument against internationally recognized screening tools in multicenter and multilingual studies, with the aim of expanding its applicability and supporting its formal adoption in global clinical and educational practice.

Implementing these directions will help refine multidimensional risk models and facilitate robust, internationally applicable protocols for early identification and intervention in dyslexia and related learning disorders.

## 5. Conclusions

This research aimed to investigate the prevalence of dyslexia-dysgraphia risk among school-aged children and its correlations with orofacial morphological features, occlusal anomalies, and neuromuscular dysfunctions. The study sought to strengthen the interdisciplinary framework between educational screening, dental diagnostics, and myofunctional therapy in addressing early indicators of language-related learning disorders.

Our findings confirm that dyslexia-dysgraphia risk is not only a cognitive or linguistic issue but often reflects measurable anatomical and functional characteristics. Statistically significant correlations with malocclusion and lip posture reinforce the need to embed orofacial assessment into early school health protocols. The absence of significant age-related variation in risk further highlights the developmental stability of these associations at the ages screened.

In clinical and educational contexts, this study advocates for the following:

- The integration of dental and orthodontic evaluations into early childhood screening.
- Increased awareness and training for educators and pediatricians in recognizing orofacial markers.
- The inclusion of multidisciplinary intervention strategies, such as orthodontic correction, logopedic therapy, and myofunctional re-education.
- A proactive approach to underdiagnosis, especially in settings with limited access to neurodevelopmental diagnostics.

Ultimately, aligning dental, cognitive, and linguistic expertise can foster more accurate diagnoses, individualized treatment pathways, and better long-term educational outcomes for children at risk of dyslexia and dysgraphia.

The present study confirms a significant link between the risk of dyslexia-dysgraphia and both neurocognitive and dental–occlusal factors in school-aged children. The findings support the following conclusions:

Dyslexia-dysgraphia affects a considerable proportion (11.14%) of children without associated speech-language disorders, underlining the need for early detection.

Malocclusion types, arch forms, and occlusal classifications correlate with increased risk, suggesting that structural orofacial anomalies may influence language acquisition and writing development.

Educational context and teacher preparedness significantly affect risk identification and outcomes.

Underdiagnosis remains a concern in some settings, emphasizing the need for systematic, interdisciplinary screening programs.

Future prevention and intervention strategies should integrate dental, neuropsychological, and educational assessments to address the multifactorial nature of these learning stomatognathic disorders.

This study contributes directly to the personalized medicine paradigm, showing that early indicators for dyslexia-dysgraphia are not limited to language or IQ metrics but are multidimensional—involving stomatognathic architecture, emotional regulation, and school context.

Going forward, this model supports the following:

Neuro-dental developmental pathways for diagnosis.

Custom therapeutic protocols (e.g., targeted tongue re-education, emotional coaching).

Informed device design (like the patented tool) adapted to specific orofacial needs.

To improve rigor in future studies, researchers might consider the following:

Collect parental education and income data and include these as covariates in statistical models.

Use standardized home language environment questionnaires.

Assess early educational history or use tools like the Early Childhood

Environment Rating Scale (ECERS).

Stratify or match participants based on SES (socioeconomic status) or language exposure to reduce bias.

## 6. Patents—Patent Information

Patent number 125059 was granted under the provisions of law No. 64/1991, republished in the Official Gazette of Romania, part i, no. 541, dated 8 August 2007. The patent covers a device designed for the stage of learning to read and write.

The inventors and patent holders are Georgeta Burlea, Anamaria Burlea, and Ștefan Lucian Burlea, all from Iași, Romania.

This patent was officially issued on 30 August 2013, in Bucharest, Romania.

## Figures and Tables

**Table 1:** School-wise distribution of screened children and identified risk.

Kindergarten	Total Children Enrolled	Children in Preparatory Group	Children with Other Language Disorders	Children Identified as at Risk for Dyslexia
GPP NR. 23—IAȘI	116	70	12	4
GPP NR. 4—IAȘI	84	23	8	0
GPP NR. 3—IAȘI	170	87	14	5
GPN NR. 27—IAȘI	169	120	20	6
GPP NR. 21—IAȘI	99	54	13	2
GPN NR. 23—IAȘI	276	102	28	2
GPN NR. 7—IAȘI	164	21	19	1
GPN NR. 25—IAȘI	150	37	23	4
GPN NR. 24—IAȘI	242	71	18	2
GPN NR. 15—IAȘI	159	63	18	4
GPP TÂRGU FRUMOS	185	125	22	2
Total	1814	773	195	32

**Table 2:** Descriptive statistics.

Outcome	Frequency	Percent (%)	Valid Percent (%)	Cumulative Percent (%)
confirmed dyslexia-dysgraphia	23	67.65	74.19	74.19
other language disorders	8	23.53	25.81	100.00
missing cases (relocated)	1	3.13	—	—
total identified	32	100.00	—	—

**Table 3:** Gender distribution (study group: n = 31; control group: n = 29).

Gender: Study Groups:	Male		Female		Total		Chi2-Test (χ^2^)	*p*-Value	Significance
	n1	%	n2	%	n	%			
cases group	19	61.29	12	38.71	31	60.00	0.0445	0.832937	NS
control group	17	58.62	12	41.38	29	40.00			
total	36	60.00	24	40.00	60	100.00			

**Table 4:** Age distribution (study group: n = 31; control group: n = 29).

Age: Study Groups:	5 Years		6 Years		7 Years		Total		Chi^2^-Test (χ^2^)	*p*-Value	Significance
	n1	%	n2	%	n3	%	N	%			
Cases group	10	32.26	14	45.16	7	22.58	31	60.00	0.3604	0.835103	NS
Control group	10	34.48	11	37.93	8	27.59	29	40.00			
Total	20	33.33	25	41.67	15	25.00	60	100.00			

**Table 5:** Comparative analysis of orofacial features by group.

Feature	Study Group Total (n=31)	Study Males (n = 19)	Study Females (n = 12)	Control Group Total (n = 29)	Control Males (n = 17)	Control Females (n = 12)	Chi2-Test	*p*-Value/Significance	Odds Ratio
Dental Modifications (DMs)	15 (48.39%)	11 (57.89%)	4 (33.33%)	9 (31.03%)	6 (35.29%)	3 (25.00%)	1.8799	0.170349/NS	2.08
Lip Disorders (LDs)	12 (38.71%)	7 (36.84%)	5 (41.67%)	3 (10.34%)	2 (11.76%)	1 (8.33%)	6.4294	0.011225*	5.47
Tongue Dysfunction (TD)	14 (45.16%)	10 (52.63%)	4 (33.33%)	0 (0.00%)	0 (0.00%)	0 (0.00%)		-	-
Occlusal Alteration (OA)	19 (61.29%)	12 (63.16%)	7 (58.33%)	8 (27.59%)	5 (29.41%)	3 (25.00%)	6.877	0.008731*	4.16
Emotional Dysregulation (ED)	17 (54.84%)	11 (57.89%)	6 (50.00%)	10 (34.48%)	6 (35.29%)	4 (33.33%)	2.5085	0.113233/NS	2.31

**Table 6:** Comparative analysis of orofacial features by gender (extended).

Feature	m Cases (n = 19)	f Cases (n = 12)	m Control (n = 17)	f Control (n = 12)	Chi2 m- vs. m	Chi2 f vs. f	*p*-Value m	*p*-Value f	Odds Ratio m	Odds Ratio f
Dental Modifications (DM)	11 (57.89%)	4 (33.33%)	6 (35.29%)	3 (25.00%)	1.8388	0.06664	0.175087/NS	0.796602/NS	2.52	1.5
Lip Disorders (LDs)	7 (36.84%)	5 (41.67%)	2 (11.76%)	1 (8.33%)	0.0095	0.75	0.923096/NS	0.386476/NS	4.38	7.86
Tongue Dysfunction (TD)	10 (52.63%)	4 (33.33%)	0 (0.00%)	0 (0.00%)			-/NS	-/NS	-	-
Occlusal Alteration (OA)	12 (63.16%)	7 (58.33%)	5 (29.41%)	3 (25.00%)	1.3716	2.7429	0.241529/NS	0.976900/NS	4.11	4.2
Emotional Dysregulation (ED)	11 (57.89%)	6 (50.00%)	6 (35.29%)	4 (33.33%)	1.8388	0.6857	0.175087/NS	0.407629/NS	2.52	2

**Table 7:** Significance and interpretation of orofacial features by group and gender.

Feature	χ^2^ Value	*p*-Value	Odds Ratio (m/f)	Interpretation
Dental Modifications (DM)	1.84 (m)/0.07 (f)	0.175/0.797	2.52/1.5	Not statistically significant; moderate increased risk in males (or = 2.52), and weak association in females; suggestive of structural involvement in certain subgroups.
Lip Disorders (LD)	0.01 (m)/0.75 (f)	0.923/0.386	4.38/7.86	Not statistically significant, but clinically suggestive; high ORs indicate possible relevance of lip dysfunction as a secondary indicator—especially in females.
Tongue Dysfunction (TD)	–	–	–	Very strong clinical association, exclusive to the study group (0% in controls); statistically untestable but highly indicative of risk.
Occlusal Alteration (OA)	1.37 (m)/2.74 (f)	0.242/0.977	4.11/4.2	Not statistically significant, but high ORs suggest a potential structural-functional marker; needs further study with larger samples.
Emotional Dysregulation (ED)	1.84 (m)/0.69 (f)	0.175/0.408	2.52/2.0	Clinically moderate association, but statistically non-significant; emotional regulation issues may be part of a broader psycho-behavioral profile in at-risk children.

**Table 8:** Pearson correlations between orofacial and emotional variables.

Variable Pair	Pearson r	Significance
Tongue Dysfunction vs. School Avoidance	0.76	*p* < 0.01 SS
Occlusal Alteration vs. Emotional Distress	0.64	*p* < 0.05 SS

**Table 9:** Comparison of emotional regulation scores.

Group	Mean ± SD	*p*-Value
Study group	3.2 ± 1.1	
Control group	4.7 ± 0.9	<0.01

**Table 10:** Logistic regression output for predictors of dyslexia risk.

Predictor	Odds Ratio (OR)	*p*-Value	χ^2^ Value	Interpretation
Age (Continuous)	1.02	0.83	0.046	Not statistically significant. OR = 1.02. No meaningful statistical or clinical effect observed. A negligible effect of age on dyslexia risk. Age alone is not a reliable predictor in this model.
Tongue Dysfunction (TD)	4.81	0.06	3.54	Borderline Significance. OR = 4.81. The predictor shows a clinically meaningful association. Individuals with tongue dysfunction are 4.8 times more likely to be at risk of dyslexia. Though *p* = 0.06 does not meet the 0.05 threshold, the effect size suggests strong clinical relevance.
Emotional Dysregulation (ED)	3.94	0.09	2.87	Borderline Significance. OR = 3.94. The predictor shows a clinically meaningful association. Children with emotional dysregulation are nearly (3,94) 4 times more likely to be at risk. While not statistically significant (*p* = 0.09), the association warrants further study in a larger sample.

## Data Availability

The data supporting the findings of this study are available in publicly accessible repositories, including Google Scholar, PubMed, and Google Search. Links to archived datasets analyzed or generated during the study are provided within the article where applicable. Additional datasets can be obtained from the corresponding authors upon reasonable request.

## Abbreviations

The following abbreviations are used in this manuscript:

Rotation (ROT)	Rotated Tooth Alignment
Vestibularized (VES)	Tooth Tilted Towards Lips/Cheeks
Palatalized (PAL)	Tooth Tilted Towards Palate/Tongue
Transposition (TRANS)	Positional Exchange Between Teeth
Prodentie (PRO)	Dental Protrusion
Procheilie (PROCH)	Lip Protrusion
Retrognathie (RETROG)	Backward Jaw Positioning
ER	Emotional Regulation
TD	Tongue Dysfunction
OA	Occlusal Alteration
DM	Dental Modifications
NDD	Neurodevelopmental Disorder
OR	Odds Ratio
Angle Class I/II/III-	Angle’s Classification of Malocclusion
	(Class I: Normal Molar Relationship;
	Class II: Distal Occlusion;
	Class III: Mesial Occlusion)
VERT	Version (Tooth Displacement in Vertical Axis)
ROT	Rotation (Rotated Tooth Alignment)
VEST	Vestibularized (Tooth Inclined Toward Lips/Cheeks)
PAL	Palatalized (Tooth Inclined Toward Palate/Tongue)
TRANS	Transposition (Exchange of Positions Between Two Teeth)
PRO	Prodentie (Protrusion of Dentition)
PROCH	Procheilie (Protrusion of Lips)
RETROG	Retrognathie (Posterior Positioning of the Jaw)
SS	Stomatognathic System
ODED	Orofacial and Emotional Dyslexia Early Detection
SS*/NS	Statistically Significant/Not Significant
SES	Socioeconomic Status
ECERS	Early Childhood Environment Rating Scale
FTMDS	Functional Temporomandibular Disorders
